# Complex partial seizure with severe depression and conduct disorder in a 15 year old female adolescent: a case report

**DOI:** 10.11604/pamj.2015.22.311.6961

**Published:** 2015-11-27

**Authors:** Ikenna Desmond Ebuenyi, Uzoechi Eze Chikezie, Princewill Chukwuemeka Stanley

**Affiliations:** 1 Department of Community Medicine, Niger Delta University Teaching Hospital, Okolobiri Bayelsa State, Nigeria; 2 Department of Mental Health, Faculty of Clinical Sciences, Niger Delta University, Amassoma Bayelsa state, Nigeria

**Keywords:** Complex partial seizure, depression, Conduct disorder, adolescents

## Abstract

Complex partial seizure complicated by psychiatric comorbidities like depression and conduct disorder presents management challenges for both the physician and parents. The etiology of such psychiatric comorbidities may be related to the seizure or to several other unrelated risk factors. The psychiatric comorbidities and the seizure affects the child's activities of daily living and are often a source of worry to parents and school authorities A high index of suspicion and a multidisciplinary approach are essential in the management of the affected adolescents.

## Introduction

Seizures are symptoms due to abnormal discharges from neurons in the central nervous system [1]. Complex partial seizures are focal seizures lasting 30 seconds to 2 minutes and characterized by impaired conciuosness [1, 2]. It represents temporal lobe epilepsy and its etiology in adolescents is often related to trauma, genetic and infective factors and brain tumor [2, 3]. Childhood epilepsy is a particular concern to psychiatrists because it is often associated with behavioral problems [1, 2]. Studies have noted the occurrence of psychiatric comorbidities with epilepsy [1, 3, 4]. The psychiatric comorbidities include depression (36.4%) [4], anxiety disorders (15-50%) [5, 6], attention-deficit hyperactivity disorder (ADHD) (29.1%) [4] and conduct disorder [3, 7]. It is pertinent to note that focal epilepsy has been significantly found to be more frequent in children and adolescents with psychiatric disorders [4]. The psychiatric comorbidities are significantly associated with age and ADHD has been found to be commoner in children while depression occurs more in adolescents [1, 4, 7].

The actual etiology of these psychiatric comorbidities is still controversial and demographic and biological factors have been identified as risk factors [1, 3–6]. Central nervous system disorder have been identified as a major risk factor and may or may not be related to the epilepsy. Social problems such as family factors have also been known to contribute to depression [4]. Irrespective of the etiology, complex partial seizure in addition to the psychiatric comorbidities affect a child's quality of life [1–6]. They disrupt the child's academic activities and are major sources of worry for parents with accompanying financial burden [1–6]. A multidisciplinary approach is essential in the overall management of complex partial seizures and associated psychiatric comorbidities. Primary health care providers and physicians should be on the lookout for psychiatric comorbidities in adolescents that present with complex partial seizures.

## Patient and observation

PE is a 15year old female student who was admitted via the accident and emergency department of the Niger Delta University Teaching Hospital (NDUTH) Yenagoa on account of loss of consciousness and irrational talk four hours prior to presentation. Four hours prior to presentation patient had frontal headache which was severe for which she went to a neighbor's house to obtain help since her parents were not at home. At the neighbor's house she was yet to obtain the medications when she fainted and lost consciousness. The duration of loss of consciousness could not be quantified but she regained consciousness without intervention but had visual and auditory hallucinations. There was no fecal or urinary incontinence. This was, apparently, the first episode of loss of consciousness and hallucinations in the patient. However, she had similar episodes of headache in the past and they often coincided with her menstrual flow. She was still menstruating when the latest incident occurred.

There was no prior history of trauma to the head, ingestion of poisonous substances or hallucinogens. Her last meal was two hours prior to the incident. There was no prior history of blood transfusion, hospital admission or mental illness in the family. Developmental history or history of febrile illness could not be ascertained.

She is the first child of her parents; mother is separated from the father. She lives with her father, step mother and 3 younger siblings from the step mother. There is history of unruly behavior, stubbornness, truancy, destructiveness, school refusal and poor performance in school. History of sexual activity could not be ascertained from both parents. There was recent associated history of low mood, social withdrawal, expression of suicidal intents and unprovoked weeping.

At presentation to the hospital, she was conscious but restless and agitated. She was afebrile (36.8°C), not pale, anicteric, not dehydrated and had no pedal edema. Her pulse rate was 89 per minute (full volume and regular), her respiratory rate was 42 cycles per minute while her blood pressure was 120/80mmHg. Mental state examination revealed a poorly dressed young female, agitated and restless. Orientation, Judgment and insight could not be ascertained. Her mood was sad and there was associated crying spells.

An assessment of Acute Psychosis? Cause was made by the Medical team on call. She was given intramuscular fluphenazine deconate 25mg stat, intravenous diazepam 10mg stat, tablets risperidone 1mg daily, tablets amitriptyline 25mg nocte. The following investigations were requested, namely full blood count, urinalysis, blood chemistry, retroviral screening malaria parasite test,widal test and electroencephalogram(EEG). All the investigations were normal with the exception of EEG which was not done due to financial constraint. She was transferred to the pediatrics unit.

Three days later, patient was still severely depressed. She was reviewed by the consultant psychiatrist who made an assessment of Complex partial seizure with severe depression and Conduct disorder. The tablets amitriptyline and risperidone were discontinued while patient was commenced on tablets carbamazepine (slow release) 200 mg two times daily and tablets sertraline 50 mg daily. She had significant reduction in depressive symptoms and was seizure free after 3 weeks on admission. She was discharged subsequently on request of her father and placed on outpatient clinic follow up.

## Discussion

Complex partial seizure was in 1981 classified as focal seizure with impaired consciousness by the International League Against Epilepsy (ILAE) [8]. Our patient was recorded as having suffered loss of consciousness, although the duration could not be quantified. The characteristic headache that preceded the seizure and subsequent irritability and change in personality has been documented in other studies [2, 9].

History and laboratory investigations did not clearly implicate trauma, infective or familial causes in the trace for etiology or triggers. Financial constraint prevented electroencephalogram (EEG), although the result is often normal [2]. Lack of universal coverage and financial constraint are major challenges to health care in low and middle income countries. Our patient had sustained features of depression and history revealed poor performance at school as well as conduct disorder. These psychiatric comorbidities coexisting with the complex partial seizure are consistent with findings in literature [1, 3–6].

## Conclusion

Psychiatric disorders are quite common among children and adolescents and most cases go undiagnosed. Epilepsies are also common among them and complex partial seizure frequently presents with psychiatric symptomatology. Presence of these comorbidities usually impair quality of life and social growth of children and adolescents especially in the developing world. As such a high index of suspicion should be held by physicians managing seizure disorders among the young. Early detection and prompt interventions will go a long way to improving the outcome of these disorders.
